# Characterization of the Effect of α-Lipoic Acid in Human Macrophages Infected with *Mycobacterium tuberculosis*

**DOI:** 10.3390/ijms27115053

**Published:** 2026-06-03

**Authors:** Alessandro Salustri, Gioia Cappelletti, Flavio De Maio, Youqing Shen, Filomena Nuzzi, Ivana Palucci, Francesco Paglione, Maurizio Sanguinetti, Michela Sali, Giovanni Delogu

**Affiliations:** 1 Department of Basic Biotechnological Sciences, Clinical, Intensive and Perioperative, Section of Microbiology, Catholic University of Sacred Heart, Largo Francesco Vito 1, 00168 Rome, Italy; gioia.cappelletti@unicatt.it (G.C.); f.nuzzi@student.unisi.it (F.N.); ivana.palucci@unicatt.it (I.P.); francesco.paglione01@icatt.it (F.P.); maurizio.sanguinetti@unicatt.it (M.S.); michela.sali@unicatt.it (M.S.); 2 Department of Laboratory and Hematologic Sciences, Fondazione Policlinico Universitario A. Gemelli IRCCS, Largo Agostino Gemelli 8, 00168 Rome, Italy; flavio.demaio@unicatt.it; 3 Microbiota Analysis & Microbial WGS Research Core Facility, GSTeP, Fondazione Policlinico Universitario A. Gemelli IRCCS, 00168 Rome, Italy; 4 Zhejiang Key Laboratory of Smart Biomaterials, Center for Bionanoengineering, College of Chemical and Biological Engineering, Zhejiang University, Hangzhou 310058, China; shenyq@zju.edu.cn; 5 Department of Medicine and Surgery, Università degli Studi di Parma, Via Gramsci 14, 43126 Parma, Italy; giovanni.delogu@unipr.it

**Keywords:** tuberculosis, host-directed therapies, α-lipoic acid, phagosome acidification, THP-1 macrophages

## Abstract

Tuberculosis (TB) treatment is severely hampered by the rise in multi-drug-resistant strains and the prevalence of drug-induced toxicities. Host-Directed Therapies (HDTs) have emerged as a promising strategy to overcome these challenges by modulating innate immunity and circumventing *Mycobacterium tuberculosis* (Mtb) evasion mechanisms. A hallmark of Mtb pathogenesis is the arrest of phagosome maturation and the induction of host cell necrosis over protective apoptosis. In this study, we investigated the potential HDT effects of α-Lipoic acid (α-LA), a well-known antioxidant and metabolic cofactor, within an in vitro model of Mtb-infected THP-1 macrophages. Our findings indicate that α-LA treatment modulates the macrophage redox state and selectively promotes apoptosis in infected cells without increasing necrotic lysis. Furthermore, α-LA administration led to a significant, dose-dependent restoration of phagolysosome acidification, effectively reversing the maturation blockade imposed by Mtb. Notably, this enhanced acidification inversely correlated with intracellular bacterial survival. These results suggest that α-LA might act as a multifaceted HDT agent capable of restoring both host-protective cell death and phagosomal microbicidal mechanisms. Given its established safety profile and its ability to complement standard anti-TB drugs like Bedaquiline (BDQ), α-LA represents a highly promising candidate for adjunct therapy to improve TB treatment outcomes and mitigate the impact of antibiotic resistance.

## 1. Introduction

Tuberculosis (TB) remains the world’s leading cause of death from a single infectious agent and is the most critical global public health challenge [1]. Progress in fighting TB is hampered by two issues: the emergence of multi-drug-resistant (MDR) and extensively drug-resistant (XDR) strains that make standard treatments ineffective, extending the duration of the treatment itself, increasing side effects and healthcare costs [2,3]; and the lack of an effective vaccine, since BCG is ineffective in preventing TB disease in adults [4,5,6].

Current anti-TB treatment relies on the administration of multiple drugs in regimens that range from six months to more than one year in the case of MDR- or XDR-TB [7,8]. Moreover, regimen duration and complexity can lead to chronic toxicity due to drug accumulation, significantly impacting patient adherence to therapy, and often driving the development of further resistance [9,10]. To overcome these limitations, research is increasingly focusing on novel therapeutic approaches, particularly Host-Directed Therapies (HDTs). Unlike antibiotics, which directly target the bacterium, HDTs are designed to boost the host’s immune response or modulate the cellular environment, with the aim of potentially shortening treatment duration, mitigating tissue damage, and enhancing drug efficacy, offering a promising strategy toward more efficient disease control [11,12,13,14].

A hallmark of Mtb pathogenesis is its ability to arrest phagosome maturation and acidification, effectively avoiding its elimination and regulating the host immune response; therefore, restoring or modulating this process appears to be a potential strategy to restore host immunity. α-Lipoic Acid (α-LA), an essential antioxidant, represents a potential candidate for this approach due to its anti-inflammatory and immunomodulatory properties [15,16,17]. α-LA functions as an essential cofactor for mitochondrial α-ketoacid dehydrogenases, playing a pivotal role in cellular energy metabolism and redox homeostasis [18]. Furthermore, as a naturally occurring compound widely used in clinical settings—such as for the treatment of diabetic neuropathy—it exhibits a very high safety profile and excellent tolerability, even during prolonged administration [19,20]. Given this solid metabolic rationale and its lack of significant cellular toxicity, this work aimed to evaluate the potential protective effects of α-LA during TB treatment by characterizing its impact on Mtb-infected human macrophages. Specifically, we investigated the hypothesis that α-LA—either alone or in combination with BDQ—could function as an HDT agent by modulating macrophage functions. The study focuses on its potential to restore the phagosomal environment and reverse Mtb-mediated maturation arrest, thereby enhancing host innate defense mechanisms in vitro.

## 2. Results

### 2.1. α-LA Exerts a Direct Anti-Mycobacterial Effect Against Mtb

To assess the direct anti-mycobacterial effect of α-LA against Mtb, we performed a broth micro-dilution assay by incubating the Mtb reference strain H37Rv with serial dilutions of α-LA (from 1 mg/mL, 4846.6 μM, to 0.003 mg/mL, 18.93 μM). This extensive range was chosen to perform an unbiased screening, as the specific inhibitory threshold of α-LA against Mtb had not been previously established. Starting from a high dosage ensured the detection of any potential direct antimicrobial activity, while the serial dilution allowed for precise identification of the Minimum Inhibitory Concentration (MIC) and for observation of a potential dose-dependent response. BDQ was used as the positive control, while untreated bacteria were used as the negative control.

**Figure 1:**
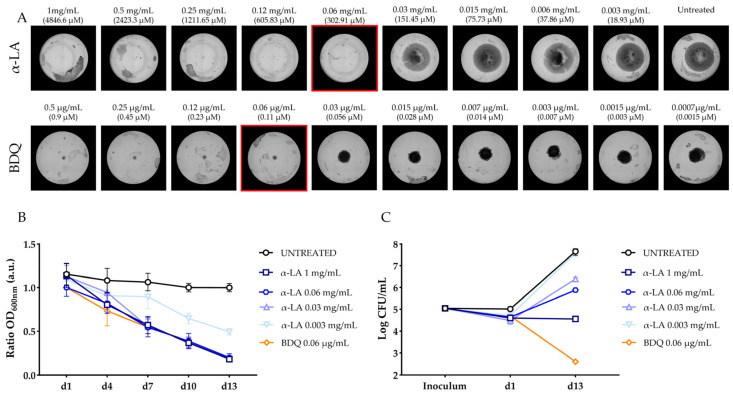
Characterization of α-LA activity in Mtb axenic culture: (**A**) Representative image of the broth microdilution assay for α-LA against Mtb H37Rv. Red boxes indicate the concentration of 0.06 mg/mL, which was identified as the threshold for inhibited visible turbidity. (**B**) Growth kinetics of Mtb H37Rv, over 13 days, monitored by optical density OD600; data are normalized and expressed as the ratio relative to the untreated control (arbitrary units, a.u.). (**C**) Determination of bacterial viability quantified as Colony Forming Units (LogCFU/mL) at 1 and 13 days post-incubation. CFU counts demonstrate that α-LA lacks a direct anti-mycobacterial effect at the concentrations tested. The concentrations of α-LA shown in the graphs were selected as representative of key experimental thresholds, including the maximum tested dose, the visual inhibition threshold, the adjacent ineffective dose, and the lowest concentration tested. Data represent the mean ± SD of 3 technical triplicates from one representative experiment out of 3 independent biological replicates performed. Statistical significance was determined by one-way ANOVA followed by Dunnett’s test.

Bacterial growth was initially assessed by the presence of a bacterial growth button. As shown in Figure 1A, visual inspection suggested a threshold of visible inhibition with α-LA up to 0.06 mg/mL (302.9 µM), whereas at 0.03 mg/mL (151.5 µM), bacterial growth was similar to the untreated control. The MIC for BDQ was confirmed to be 0.06 µg/mL (0.11 µM), as reported in the literature. To further characterize this effect, we monitored growth kinetics over a 13-day period (Figure 1B). Optical density (OD_600_) data confirmed a dose-dependent reduction in turbidity. While no significant differences were observed during the early stages (days 1–4), α-LA at 0.06 mg/mL and 1 mg/mL induced a statistically significant decrease in bacterial density starting from day 7 (*p* ≤ 0.05), which optically matched the profile of BDQ. By day 13, the reduction in OD_600_ for these doses was highly significant compared to the untreated control (*p* ≤ 0.001). Crucially, however, these optical findings were not supported by the viability analysis. Determination of Colony Forming Units (in CFU/mL) revealed that α-LA does not exert a direct bactericidal effect at the tested concentrations (Figure 1C). Despite the reduction in turbidity, Mtb remained viable at 0.06 mg/mL, with CFU counts reaching approximately 10^6^ by day 13. A bacteriostatic effect was only achieved at the highest concentration (1 mg/mL). Overall, these findings highlight a significant discrepancy between optical-based measurements and actual bacterial viability, suggesting that the observed reduction in turbidity may result from non-lethal phenomena, such as bacterial aggregation or metabolic slowdown, rather than direct mycobactericidal activity.

### 2.2. α-LA Exhibits a Dose-Dependent Safety Profile in Human THP-1 Macrophages

**Figure 2:**
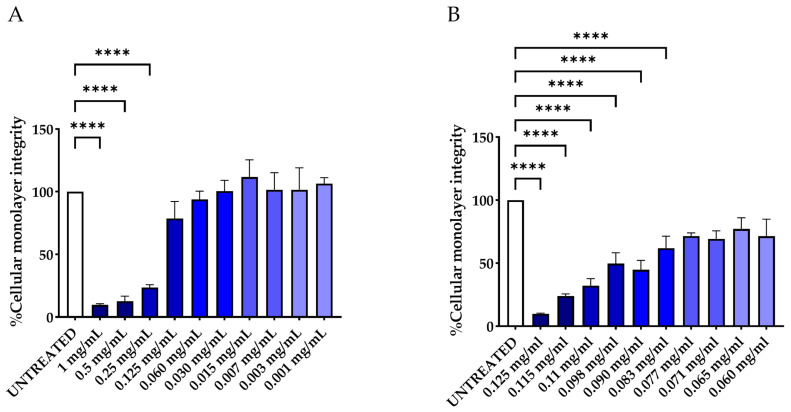
α-LA cytotoxicity screening and identification of the maximum non-toxic concentration: (**A**) Quantitative assessment of THP-1 monolayer integrity after 5 days of exposure to α-LA (1.0 to 0.001 mg/mL). (**B**) Refined screening (0.125 to 0.060 mg/mL) used to identify the optimal working concentration (0.075 mg/mL, 363.5 µM). Data are expressed as percentage viability relative to untreated controls, and data represent the mean ± SD of 3 technical triplicates from one representative experiment out of 3 independent biological replicates performed. Statistical significance was determined by one-way ANOVA followed by Dunnett’s test; **** *p* < 0.0001.

To determine the biocompatibility of α-LA on human macrophages, differentiated THP-1 cells were exposed to different concentrations. The cytotoxic effect was monitored by evaluating cellular monolayer integrity via Crystal Violet staining (Figure 2A).

Strong cytotoxicity was observed at concentrations ≥ 0.125 mg/mL. Conversely, α-LA showed no toxic effects at concentrations ≤ 0.06 mg/mL. This safety profile was consistently maintained throughout the incubation period and confirmed at the latest time point (5 days post-incubation), which was selected for quantitative assessment as it represents the most critical condition of chronic exposure (Figure 2A). To precisely identify the highest non-toxic dose, a second experiment was performed using ten narrow-range serial dilutions (0.125 mg/mL to 0.060 mg/mL) (Figure 2B). This analysis established 0.077 mg/mL as the concentration at which the cellular monolayer remained statistically indistinguishable from the untreated control. Consequently, a concentration of 0.075 mg/mL was selected as the optimal working concentration for all subsequent in vitro infection assays. This dose was chosen to maximize the potential immunomodulatory effects of α-LA while ensuring the total preservation of host cell homeostasis.

### 2.3. α-LA Restricts Mtb Intracellular Growth in THP-1 Macrophages

**Figure 3:**
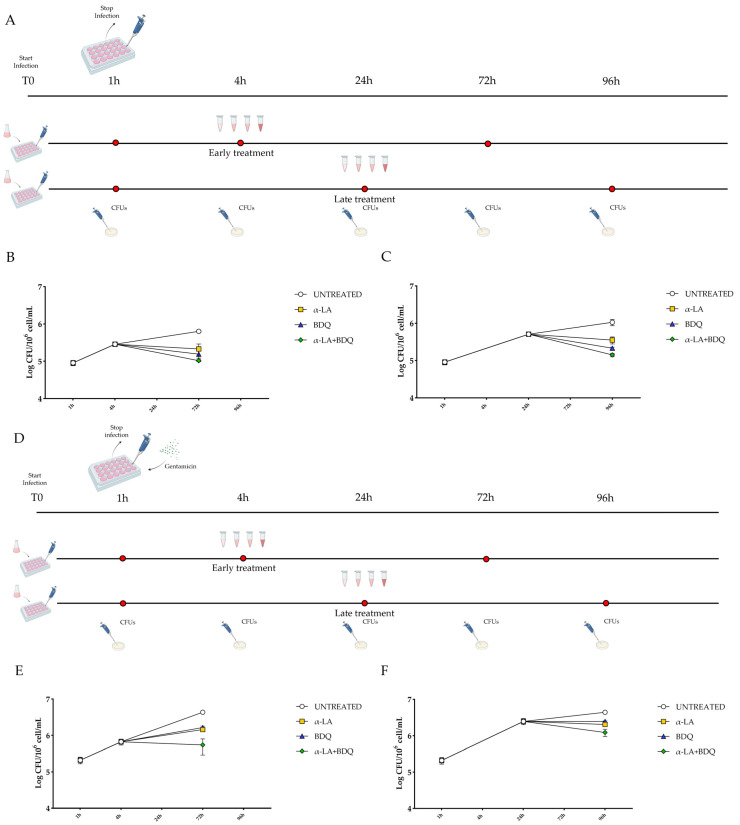
Evaluation of the intracellular anti-mycobacterial effect of α-LA in THP-1 macrophages: (**A**,**D**) Time-course evaluation of intracellular Mtb H37Rv survival (MOI 1:1) treated with α-LA (0.075 mg/mL) alone or in combination with BDQ. (**B**,**C**) Survival of total bacteria. (**E**,**F**) Survival after gentamicin treatment to isolate intracellular activity. Bacterial load is expressed as LogCFU normalized to 10^6^ macrophages. Data represent the mean ± SD of 3 technical triplicates from one representative experiment out of 3 independent biological replicates performed. Statistical significance was determined by two-way ANOVA followed by Dunnett’s post hoc test.

To evaluate the effect of α-LA on Mtb-infected human macrophages, THP-1 cells were infected with the Mtb H37Rv strain, and the antimicrobial activity of α-LA was assessed in two distinct experimental settings to characterize its role throughout different stages of pathogenesis (Figure 3A):

Early-phase treatment (administered 4 h post-infection, p.i.);

Late-phase treatment (administered 24 h p.i.).

In the untreated control, Mtb exhibited a progressive increase in bacterial load, reaching the peak of infection by 96 h p.i. Results showed that both α-LA and BDQ monotherapies significantly reduced the intracellular bacterial load at 72 h p.i. (*p* ≤ 0.01 and *p* ≤ 0.001, respectively) compared to the untreated control, while the co-treatment resulted in a more pronounced decrease in CFUs (*p* ≤ 0.01). This inhibitory effect remained statistically significant (*p* ≤ 0.05) for all treatment groups at the final time point of 96 h p.i. (Figure 3C). Crucially, a comparative analysis between groups revealed that the efficacy of α-LA monotherapy was statistically comparable to that of BDQ throughout the experiment. Despite the significantly lower MIC of BDQ compared to α-LA in cell-free cultures, no significant difference in CFU reduction was observed between the two monotherapies within the macrophages. Furthermore, while the α-LA+BDQ co-treatment did not differ significantly from BDQ alone at 72 h, when administered 24 h p.i., it demonstrated a higher efficacy compared to BDQ monotherapy at 96 h p.i. (*p* ≤ 0.01). The ability of α-LA to achieve a level of intracellular control equivalent to BDQ, despite lacking a direct effect in axenic cultures, is a key finding. Indeed, this parity in potency points toward a mechanism that leverages host cell defenses to restrict Mtb replication, providing the rationale for investigating α-LA as a potential Host-Directed Therapy (HDT).

To confirm that the antimicrobial efficacy of α-LA was specifically directed against the intracellular bacterial population, a gentamicin protection assay was performed (Figure 3D). In this setting, extracellular bacilli were eliminated by gentamicin treatment (1 h p.i.) prior to the administration of treatments at 4 h and 24 h p.i. Early-phase treatment showed that at 72 h p.i., both α-LA and BDQ monotherapies significantly reduced the intracellular bacterial load compared to the untreated control (*p* ≤ 0.05). The co-treatment maintained a higher degree of efficacy in this experimental setting (*p* ≤ 0.01) compared to the untreated control. Consistent with the previous model, no statistically significant difference was observed between the effects of α-LA and BDQ monotherapies, while the co-treatment confirmed a higher effect (*p* ≤ 0.01). Notably, late-phase treatments showed that the inhibitory effect of BDQ monotherapy was no longer statistically significant compared to the untreated control at the final time point. In contrast, α-LA monotherapy sustained a reduction in bacterial load, maintaining statistical significance (*p* ≤ 0.05). The co-treatment continued to show a more marked decrease in CFUs (*p* ≤ 0.01). Furthermore, a comparative analysis between treatment groups revealed that while no significant difference was observed between the two monotherapies, the co-treatment showed a higher efficacy compared to BDQ monotherapy (*p* ≤ 0.05). These findings suggest that in this specific model of late-stage infection, α-LA provides a more stable restriction of Mtb replication compared to BDQ. This observation might support the hypothesis that α-LA may leverage host-mediated defenses to maintain control of the infection, a possibility further explored in the following sections.

### 2.4. α-LA Attenuates Mtb Infection-Induced ROS Production in THP-1 Macrophages

**Figure 4:**
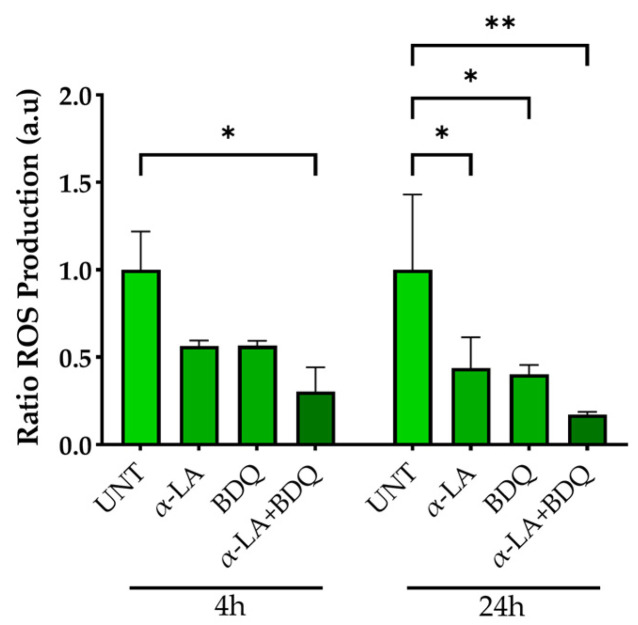
Modulation of ROS production in Mtb-infected THP-1 macrophages: Quantification of oxidative stress via CellROX Green staining 4 h and 24 h post-infection. Data are expressed as a normalized ratio (a.u.) relative to the untreated controls (UNT). Data represent the mean ± SD of 3 technical triplicates from one representative experiment out of 3 independent biological replicates performed. Statistical significance was determined by two-way ANOVA followed by Dunnett’s test (compared to respective untreated controls); * *p* < 0.05, ** *p* < 0.01.

The production of Reactive Oxygen Species (ROS) represents a fundamental but complex component of the innate immune response against Mtb [21,22]. While ROS are traditionally associated with direct mycobacterial killing, their role remains controversial: in fact, excessive oxidative stress can lead to host tissue damage and facilitate Mtb survival by promoting an inflammatory environment that impairs effective cellular responses [23,24]. Therefore, the ability of a Host-Directed Therapy (HDT) to modulate the redox state, rather than simply amplify host bactericidal pathways, might be crucial for maintaining macrophage homeostasis during infection. In this context, we evaluated the impact of α-LA—a well-documented antioxidant—on THP-1 cell ROS production during Mtb infection [17,25] (Figure 4).

The results indicated that macrophages treated 4 h p.i. with either α-LA or BDQ showed a comparable, even though not significant, reduction in ROS production compared to the untreated control. The co-treatment exhibited lower ROS levels compared to either monotherapy (*p* ≤ 0.05). In the late-treatment setting (24 h p.i.), untreated cells showed comparable levels of ROS. Cells treated with α-LA and BDQ monotherapies led to statistically significantly lower ROS levels (*p* ≤ 0.05); the co-treatment led to a further ROS reduction (*p* ≤ 0.01) (Figure 4). These findings suggest that α-LA contributes to the modulation of the macrophage redox environment. Given the controversial nature of ROS-mediated killing, the observed suppression of ROS by α-LA may not represent a reduction in microbicidal capacity, but rather an attenuation of infection-induced oxidative stress. This antioxidant activity might support host cell viability, providing a balanced environment that allows the macrophage to engage alternative, more effective antimicrobial pathways.

### 2.5. α-LA Induces Apoptosis in Infected Human THP-1 Macrophages

**Figure 5:**
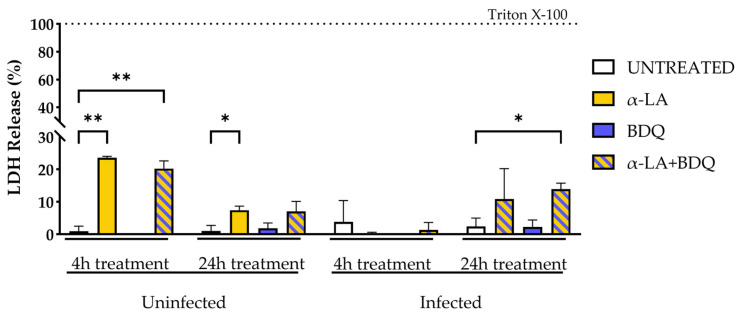
Evaluation of cell damage during Mtb infection: Quantification of LDH release into the culture supernatant as a marker of cell membrane damage. Data represent the mean ± SD of 3 technical triplicates from one representative experiment out of 3 independent biological replicates performed. Statistical significance was determined by two-way ANOVA followed by Dunnett’s multiple comparisons test (compared to the respective untreated controls); * *p* < 0.05, ** *p* < 0.01.

To determine whether the intracellular antimicrobial activity of α-LA was associated with direct host cell toxicity or regulated cellular pathways, we evaluated host cell viability by measuring the release of Lactate Dehydrogenase (LDH) in culture supernatants (Figure 5).

**Figure 6:**
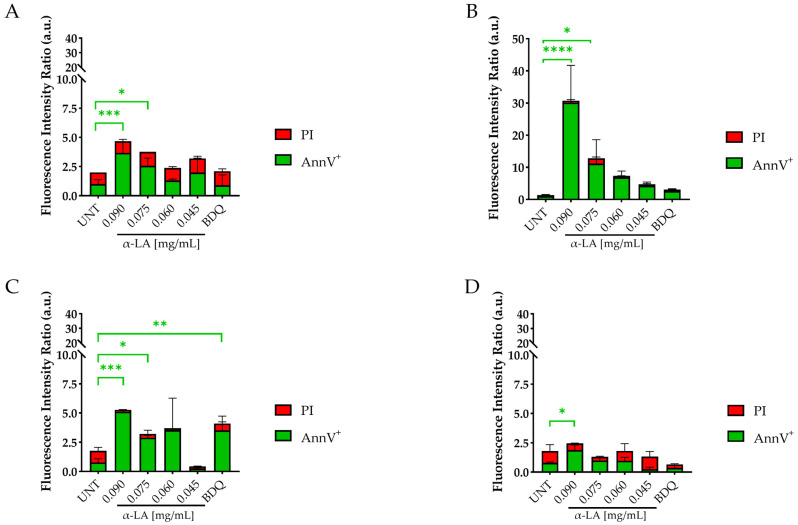
Characterization of cell death mechanisms via fluorescence microscopy assay: Apoptosis and necrosis levels were tracked by measuring the fluorescence intensity of Annexin V and Propidium Iodide in THP-1 macrophages in uninfected (**A**,**B**) and Mtb-infected cells (**C**,**D**), evaluated at 4 h and 24 h post-treatment. α-LA induces dose-dependent apoptosis without triggering necrosis in any treatment group or at any time point. Data are expressed as a normalized ratio (arbitrary units, a.u.) relative to the untreated control (UNT), and represent the mean ± SD of 3 technical triplicates from one representative experiment out of 3 independent biological replicates performed. Statistical significance was determined by two-way ANOVA followed by Dunnett’s multiple comparisons test; * *p* < 0.05, ** *p* < 0.01, *** *p* < 0.001, **** *p* < 0.0001.

In uninfected THP-1 cells, early treatment with α-LA, alone or in combination with BDQ, led to a significant increase in LDH release compared to untreated controls (*p* ≤ 0.01), reaching values of approximately 20–25%. This effect was markedly reduced when the treatments were administered at the 24 h time point (*p* ≤ 0.05 for α-LA monotherapy). A similar trend was observed in Mtb-infected macrophages. While the early treatment (4 h p.i.) showed negligible LDH levels across all groups, late-phase administration (24 h p.i.) of α-LA alone and the co-treatment resulted in an increase in LDH leakage compared to the untreated infected control (not significant and *p* ≤ 0.05, respectively). Interestingly, BDQ monotherapy did not induce significant LDH release in any of the tested conditions. These results, characterized by a controlled increase in LDH release without reaching massive lysis, appear consistent with the induction of a programmed cell death process. To further investigate this hypothesis, we characterized the impact of α-LA on the macrophage mechanism of cell death, in both uninfected and Mtb-infected macrophages, using Annexin V and Propidium Iodide stains (Figure 6).

At 4 h post-incubation, cells exhibited a distinct apoptotic phenotype (AnnV^+^) that followed a dose-dependent trend, with a statistically significant induction of programmed cell death. Specifically, treatment with α-LA at 0.090 mg/mL and 0.075 mg/mL induced a highly significant increase in programmed cell death (*p* ≤ 0.001 and *p* ≤ 0.05, respectively) compared to the untreated control (Figure 6A). A similar trend was observed in Mtb-infected macrophages at this early time point, where the 0.090 and 0.075 mg/mL groups showed a significant rise in apoptotic cells (*p* ≤ 0.001 and *p* ≤ 0.05, respectively) (Figure 6C). By 24 h post-incubation, uninfected cells exhibited a robust apoptotic response, with levels reaching approximately 30% in the 0.090 mg/mL group (*p* ≤ 0.0001) and 10% in the 0.075 mg/mL group (*p* ≤ 0.05) (Figure 6B). In contrast, Mtb-infected macrophages showed a relatively lower induction of apoptosis across all treated groups compared to their uninfected counterparts; nevertheless, the 0.090 mg/mL concentration maintained a statistically significant pro-apoptotic effect (*p* ≤ 0.05) (Figure 6D). Crucially, at both time points and across all dosages, no significant evidence of necrosis was detected, with IP signals remaining at baseline or substantially lower than the positive controls. These findings suggest that α-LA may selectively promote programmed cell death (apoptosis) in Mtb-infected macrophages without triggering necrotic cell lysis, indicating a potential mechanism to limit the spread of the pathogen.

### 2.6. α-LA Triggers Phagosome Acidification in THP-1 Macrophages

**Figure 7:**
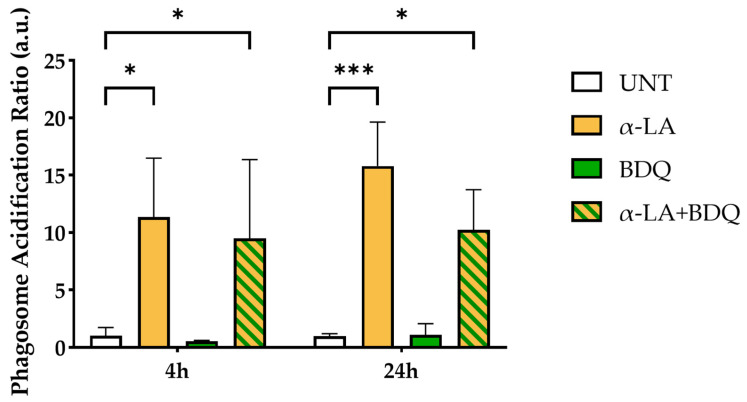
Evaluation of phagosome acidification in Mtb-infected THP-1 macrophages: Phagosome acidification was evaluated by measuring the fluorescence intensity ratio (arbitrary units, a.u.) of the intracellular pH-sensitive dye LysoTracker Yellow HCK-123. α-LA promotes phagosome acidification both alone and in combination with BDQ at 4 h and 24 h post-treatment. Data represent the mean ± SD of 3 technical triplicates from one representative experiment out of 3 independent biological replicates performed. Statistical significance was determined by two-way ANOVA followed by Dunnett’s multiple comparisons test; * *p* < 0.05, *** *p* < 0.001.

Mtb is an intracellular pathogen that persists in human macrophages by blocking phagosome–lysosome fusion and preventing subsequent acidification [26]. The arrest of phagosome maturation is a main virulence mechanism that allows Mtb to establish a stable replicative niche, thus escaping host killing [27]. Given the crucial role of phagosome maturation during Mtb infection, we investigated whether α-LA impacts phagosomal acidification. THP-1 cells were infected and treated at 4 h and 24 h post-infection (p.i.) with α-LA, BDQ, or their combination. Phagosome acidification was assessed by quantifying green fluorescence using a pH-sensitive fluorescent probe (Figure 7).

Cells receiving the early treatment (4 h p.i.) with α-LA and α-LA/BDQ exhibited a significant increase in fluorescence compared to untreated and BDQ-treated cells (*p* ≤ 0.05). Notably, BDQ alone did not alter acidification status, showing levels comparable to the untreated control. A similar, slightly more pronounced effect was observed when treatments were administered at the later time point (24 h p.i.). Under these conditions, α-LA alone (*p* ≤ 0.001) and the co-treatment (*p* ≤ 0.05) significantly enhanced phagosomal acidification compared to the untreated control. Collectively, these data suggest that α-LA might promote phagosomal acidification, regardless of whether the treatment is initiated early or late post-infection.

**Figure 8:**
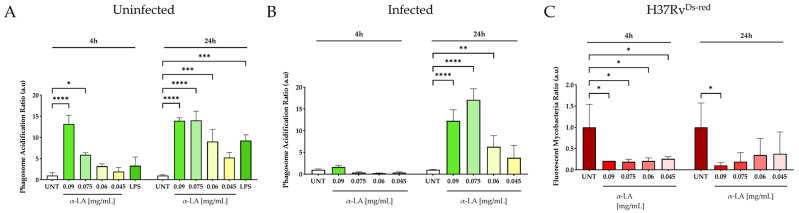
Evaluation of phagosome acidification and bacterial fluorescence in THP-1 macrophages treated with serial dilutions of α-LA: Phagosome acidification was assessed in uninfected (**A**) and Mtb-infected (**B**) cells at 4 h and 24 h post-treatment by measuring the fluorescence intensity ratio (arbitrary units, a.u.) of LysoTracker Yellow HCK-123. Intracellular bacterial presence was monitored via quantitative fluorescence microscopy of the red-fluorescent H37Rv^Ds-red^ strain (**C**). Untreated cells (UNT) and LPS-treated cells served as negative and positive controls, respectively. α-LA promotes a dose-dependent increase in phagosome acidification that correlates with a concurrent reduction in mycobacterial fluorescence. Data represent the mean ± SD of 3 technical triplicates from one representative experiment out of 3 independent biological replicates performed. Statistical significance was determined by two-way ANOVA followed by Dunnett’s multiple comparisons test; * *p* < 0.05, ** *p* < 0.01, *** *p* < 0.001, **** *p* < 0.0001.

To further investigate the observed effect of α-LA on phagosome acidification, we infected THP-1 macrophages with Mtb strain H37Rv expressing the fluorescent protein Ds-red Mcherry. Cells were treated with scalar concentrations of α-LA, while untreated cells were used as the negative control, and cells stimulated with LPS were used as the positive control (Figure 8).

At 4 h post-treatment, uninfected cells showed a dose-dependent increase in fluorescence, indicating phagosome acidification. This effect was highly significant at 0.090 mg/mL (*p* ≤ 0.0001) and 0.075 mg/mL (*p* ≤ 0.05) compared to untreated controls. The effect was further confirmed at 24 h post-infection, with significant fluorescence levels observed across all treated groups (*p* ≤ 0.0001 for 0.090 and 0.075 mg/mL; *p* ≤ 0.001 for 0.060 mg/mL) (Figure 8A). Infected cells initially displayed a lower baseline of phagosomal acidification at 4 h p.i., consistent with the well-characterized Mtb-dependent maturation block. While a dose-dependent trend was visible at this early stage, no statistically significant difference was observed between the treated and untreated infected groups. However, by 24 h p.i., α-LA displayed a robust and significant increase in phagosome acidification (*p* ≤ 0.0001 for 0.090 and 0.075 mg/mL; *p* ≤ 0.01 for 0.060 mg/mL) (Figure 8B). Notably, the acidification levels in infected macrophages at 24 h were comparable to those observed in their uninfected counterparts. Complementary evaluation of bacterial survival, monitored via DsRed-mCherry fluorescence, revealed an inverse correlation with acidification. Specifically, higher α-LA concentrations—which promoted the strongest phagosomal acidification—correlated with a significant reduction in bacterial red fluorescence (*p* ≤ 0.05); conversely, lower doses of α-LA were associated with increased bacterial persistence (Figure 8C). Collectively, these results suggest that the induction of phagosomal acidification is a pivotal mechanism through which α-LA counteracts Mtb survival strategies, promoting intracellular pathogen clearance.

## 3. Discussion

TB remains a leading cause of death worldwide from a single infectious agent. The rise in drug-resistant strains and the lack of an effective vaccine necessitate the development of novel therapeutic strategies, such as HDTs, aimed at enhancing the host’s immune response. In this study, we investigated the immunomodulatory and antimycobacterial effects of α-LA in a human macrophage model of Mtb infection.

A key observation of this study is the sustained restriction of Mtb intracellular growth provided by α-LA. Notably, the gentamicin protection assay revealed that while the inhibitory effect of the BDQ failed to maintain CFU reduction at the final time point of 96 h p.i., α-LA monotherapy sustained its antimycobacterial effect in bacterial load. This divergence is particularly interesting given α-LA’s higher MIC in cell-free broth. This reversal of relative potency within the intracellular environment suggests a mechanism that leverages host cell defenses rather than relying solely on direct bactericidal activity, a hallmark of an effective HDT agent.

Regarding the host’s physiological response, α-LA administration led to a measurable suppression of infection-induced ROS production; however, the interpretation of this reduction requires caution. As highlighted by the complex role of ROS in human antimycobacterial immunity, oxygen radicals may not be the primary effector for Mtb killing in humans [23], and the pathogen has evolved robust transcriptional responses to resist oxidative stress [21].

Our findings suggest that the observed reduction in ROS levels by α-LA might represent an attenuation of detrimental oxidative stress rather than a compromised host’s microbicidal capacity. Excessive ROS accumulation is known to trigger bivalent host responses, including harmful pathways like necroptosis, which can facilitate bacterial spread [22]. Therefore, by mitigating this oxidative damage, α-LA may support host cell viability and homeostasis, creating a balanced environment that allows the macrophage to engage more effective antimicrobial pathways.

This hypothesis might explain our results regarding phagosome maturation. The intracellular survival of Mtb is intrinsically linked to its ability to manipulate phagosome maturation by preventing its fusion with the lysosome [28,29,30]. This blockage is crucial for establishing a stable, non-acidic replicative niche [26,27]. Our data suggest that α-LA treatment may allow infected macrophages to actively overcome this hindrance, triggering a dose-dependent phagosome acidification. This effect, which was not observed with BDQ monotherapy, suggests that α-LA might support the restoration of a fundamental innate defense mechanism. The inverse correlation between increased acidification and reduced bacterial survival—respectively observed through the increase in green fluorescence of phagosomes probed with pH-responsive dye and the decrease in red fluorescence of Mtb-expressing fluorescent protein Ds-red mCherry—hints at a role for phagosome maturation in α-LA-mediated Mtb control.

Furthermore, the struggle for control over macrophage fate is a critical determinant of TB progression. Scientific consensus holds that apoptosis is a death mechanism favorable for the host, effectively restraining infection dissemination and limiting pathogen spread by allowing the contained clearance of infected cells [31,32]. Conversely, necrosis is the Mtb-favorable pathway, resulting in the uncontrolled release of viable bacilli that promote bacillary and disease spread [33,34,35]. Our results suggest that α-LA selectively promotes apoptosis in Mtb-infected THP-1 macrophages without increasing necrotic lysis. This shift toward programmed cell death, possibly linked to the modulation of the intracellular redox environment, suggests that α-LA may reprogram the host cell to overcome Mtb pro-necrotic evasion strategies. Although limited to a single cell line and a specific Mtb strain, our findings pave the way for a broader characterization of α-LA’s role, which should be further validated in more complex infection models, such as primary human cells and in vivo systems.

## 4. Materials and Methods

### 4.1. Bacterial Manipulation

Mtb reference strain H37Rv was grown in 7H9 broth medium (Difco, Sparks, MD, USA) enriched with 10% albumin dextrose catalase (ADC) (Sigma-Aldrich, Schnelldorf, Germany) and 0.05% Tween 80 (Sigma-Aldrich, Schnelldorf, Germany), without antibiotics, at 37 °C, and with 110 rpm agitation. When bacterial culture reached an OD_600nm_ ~0.6, it was supplemented with 20% sterile pure glycerol (Carlo Erba Reagents, Cornaredo, Italy), and aliquots were stored at −80 °C. All experiments involving Mtb manipulation were performed at a Biosafety level 3 laboratory (BSL3) at the Institute of Microbiology of Policlinico Universitario Agostino Gemelli—IRCCS [36].

### 4.2. Cellular Manipulation

Human monocyte-derived macrophages, THP-1 cells, were grown in Roswell Park Memorial Institute (RPMI) 1640 medium (Euroclone, Pero, Milano, Italy) complemented with 10% Fetal Bovine Serum (Corning), 1% L-Glutamine, and 1% penicillin/streptomycin and were incubated in 5% CO2 atmosphere, 80% humidity, and at 37 °C. For in vitro infection experiments, THP-1 cells were harvested and centrifuged at 1000 rpm for 10′, at room temperature. Supernatant was discharged, and cell pellets were resuspended in RPMI complemented with 5% Fetal Bovine Serum, 1% L-Glutamine (Euroclone, Pero, Milano, Italy), without antibiotics, and with Phorbol Myristate Acetate (PMA) (Sigma-Aldrich, Schnelldorf, Germany) at a final concentration of 10 ng/mL to induce differentiation, plated at a concentration of ~10^6^ cells/mL/well. After 24 h, medium containing PMA was removed, and cells were washed with PBS prior to infection and/or treatments [37].

### 4.3. Minimum Inhibitory Concentration Assay

MIC determination was conducted by broth microdilution assay [38,39]. Briefly, α-LA was resuspended in 7H9 broth medium (Difco) enriched with 10% ADC, without Tween80, at a final concentration of 2 mg/mL. Several serial dilutions in base 2 were made up to a concentration of 0.003 mg/mL, and 50 µL of each solution was seeded in triplicate in round-bottom 96-well multi-well plate. Mtb reference strain H37Rv at a concentration of 2·10^5^ CFU/mL was plated to reach a final concentration of 1·10^5^ CFU/mL in each well containing 2x α-LA solutions. BDQ was used as the positive control [40], while untreated medium was used as the negative control. Plates were incubated in 5% CO_2_ atmosphere, 80% humidity, and at 37 °C. At 14 days post-incubation, Mtb was inactivated with 100 µL of 8% PFA for 30 min. Representative images of entire wells were taken for each condition with Cytation5 (Agilent BioTek, Winooski, VT, USA). Results were further confirmed by plating bacterial solutions on Middlebrook 7H11 (Millipore, Burlington, MA, USA) enriched with Middlebrook OADC Growth Supplement (Becton Dickinson, Sparks, MD, USA) for bacterial survival evaluation through CFU counts at 24 h and 13 days post-incubation.

### 4.4. Optical Density (OD_600nm_) Growth Assays

Bacterial growth kinetics were monitored using a broth dilution assay performed in conical tubes. Mtb H37Rv (ATCC, Manassas, VA, USA) at an initial concentration of 10^5^ CFU/mL was cultured in Middlebrook 7H9 broth as previously described. The bacterial suspension was incubated at 37 °C in the presence of serial dilutions of α-LA (1 mg/mL to 0.003 mg/mL) or BDQ (0.5 µg/mL to 0.0007 µg/mL). Growth was monitored over a 13-day period by measuring the optical density at 600 nm (OD_600nm_) using a microplate reader at specific time intervals (days 1, 4, 7, 10, and 13) [41]. For each measurement, an aliquot was transferred into a 96-well plate to allow spectrophotometric reading. Each condition was performed in triplicate. To account for the intrinsic absorbance of the compounds, background subtraction was performed using wells containing medium and the respective compound concentrations without bacteria. The Minimum Inhibitory Concentration (MIC) was defined as the lowest concentration of α-LA that led to a turbidity comparable to BDQ at its MIC point—effectively preventing any visible bacterial growth—and significant increases in OD_600nm_ compared to the initial inoculum.

### 4.5. Crystal Violet Cytotoxicity Assay

Human monocyte-derived macrophages were differentiated as described above. Cells were seeded at a concentration of 6·10^5^ cells/mL/well and incubated with α-LA at different concentrations, ranging from 1 mg/mL up to 0.001 mg/mL, in 5% CO_2_ atmosphere, 80% humidity, and at 37 °C. At 6 h, 24 h, 48 h, 72 h, 96 h, and 120 h post-incubation, medium was removed, and cells were stained with Crystal Violet solution (PREVI COLOR GRAM—Biomerieux, Marcy-l’Étoile, France). After 15′ incubation time, Crystal Violet solution was removed, and cell pellets were washed with ddH_2_O until the washes became transparent. Color brightfield photos of entire wells were acquired with Cytation5 (Agilent-Biotek, Winooski, VT, USA) to evaluate α-LA cytotoxicity through cellular monolayer integrity [42]. A second experiment with intermediate dilutions ranging from a maximum concentration of 0.125 mg/mL to a minimum concentration of 0.060 mg/mL, with a dilution factor of 1.076, was performed to identify the highest non-toxic concentration to be used in in vitro infection experiments.

### 4.6. In Vitro Infection Model

THP-1 cells were differentiated and plated as described in Section 2.2. Cells were infected with Mtb reference strain H37Rv at an MOI of 1:1. At 1 h p.i., infective solution was removed, and cells were washed with PBS and rinsed with fresh medium. Intracellular bacterial load was monitored by CFU counts at 1, 4, and 24 h p.i. to establish the baseline of infection, and subsequently at 72 (early-phase treatment) and 96 h p.i. (late-phase treatment) to evaluate treatment efficacy. To strictly evaluate intracellular anti-mycobacterial activity, in a separate set of experiments, extracellular bacteria were eliminated by incubating cells with 50 µg/mL gentamicin (Sigma-Aldrich) for 1 h, followed by three washes with PBS prior to treatment. In both experimental settings, to mimic early and late stages of infection, respectively [43,44,45], cells were treated at 4 h and 24 h post-infection with 0.075 mg/mL α-LA, alone or in combination with BDQ at its MIC point. At 3 days and 4 days post-infection, supernatants were removed and stored at −80 °C, and further assayed for LDH release. Cells were washed with PBS and lysed with Triton X-100 at 0.1%. Lysed cell solutions containing intracellular bacteria were plated on Middlebrook 7H11 enriched with Middlebrook OADC Growth Supplement (Becton Dickinson, Sparks, MD, USA) for bacterial load evaluation through CFU counts.

### 4.7. ROS Production

THP-1 cells seeded at a concentration of ~1·10^5^ cells/mL/well were plated and differentiated as described above. After 24 h, medium was removed, and cells were infected with Mtb reference strain H37Rv, with an MOI of 1:1. At 1 h p.i., the infection was stopped, and the infective solution was replaced with fresh medium. α-LA at 0.075 mg/mL was added 4 h p.i. and 24 h p.i., alone and in combination with BDQ at the MIC point. At 3 days p.i., supernatants were removed, and cells were incubated with 5 µM CellROX^®^ Green Oxidative Stress Reagent (cat. C10444, Molecular Probes, Life technologies, Carlsbad, CA, USA) resuspended in RPMI culture medium with 5% FCS and 1% L-Glutamine. At 30′ post-incubation, medium was removed, and cells were washed three times with PBS and fixed with 4% paraformaldehyde. Samples were acquired with Cytation5 in phase contrast and GFP channels with a magnification of 20× [46].

### 4.8. LDH Release Cytotoxicity Assay

Supernatants from infected THP-1 cells were harvested and stored, as described in Section 2.5. Samples were diluted at a ratio of 1:10 in fresh RPMI culture medium with 5% FCS and 1% L-Glutamine. Samples were incubated with Cytotoxicity Detection Kit (LDH) 11644793001 (Roche, Mannheim, Germany), according to manufacturer’s instructions: briefly, 0.250 mL of Solution A containing a catalyst (Diaphorase/NAD+) and 11.25 mL of Solution B containing a dye solution (INT/Na-lactate) were mixed and incubated with samples at a ratio of 1:1. At 30′ post-incubation, plate was assayed with an EL808 Microplate Reader (Biotek, Winooski, VT, USA) at 490 nm absorbance. The percentage of cell damage was calculated as: %Cell damage = $\frac{\left(Compound\;LDH\;release-Spontaneus\;LDH\;release\right)}{\left(Maximum\;LDH\;release-Spontaneous\;LDH\;release\right)}\;\times 100$, where “Spontaneus LDH release” is the value provided by untreated cells and “Maximum LDH release” is the value provided by cells treated with Triton X-100 [42,47].

### 4.9. AnnV/IP Assay

For phenotypic characterization of cell death, 10^5^ cells/mL/well were differentiated as described above and seeded in the 24-well µ-Plate 24 Well (cat. 82426, Ibidi, Gräfelfing, Germany) and infected with Mtb reference strain H37Rv as described above. Cells were treated with α-LA at 0.09, 0.075, 0.06, and 0.045 mg/mL. At 4 h and 24 h post-incubation, cells were washed with PBS and stained with ANNEXIN A5—FITC kit—IM3546 (Beckman coulter, Brea, CA, USA), according to user manual. Cells were incubated for 30′ at room temperature and were washed and rinsed with PBS. Cells treated with H_2_O_2_ 600 µM were used as the positive control, while uninfected cells were used as the negative control. Cells were assayed within 30′, as recommended by the manufacturer. Representative images of each sample were acquired with Cytation5 in brightfield: GFP channel (465 nm LED, Ex 469/35, Em 525/39 Filter Cube) for AnnV^FITC+^ apoptotic cell evaluation, and Texas Red channel (590 nm LED, Ex 586/15, Em 647/57 Filter Cube) for IP+ necrotic cell evaluation [48,49,50]. Fluorescence was quantified with Fiji software (ImageJ, V. 2.16.0/1.54p; Java 21.0.7 [64-bit]).

### 4.10. Lysosome Acidification

To evaluate lysosome acidification, cells were differentiated and seeded, as described above, at a final concentration of 10^5^ cells/mL/well. Cells were infected with Mtb reference strain H37Rv with an MOI of 1:1, and 1 h p.i., bacterial solution was removed. At 4 h p.i., cells were treated with α-LA at 0.075 mg/mL, alone or in combination with BDQ at the MIC point. Uninfected cells were used as the negative control. At 72 h p.i., supernatants were removed, and cells were washed with PBS and incubated with RPMI culture medium with 5% FCS and 1% L-Glutamine containing LysoTracker^®^ Yellow-HCK-123 (Cat. L12491, Molecular Probes, Life TechnologiesTM, Carlsbad, CA, USA), as recommended by the manufacturer [21]. Cells were incubated for 1 h, then supernatants were removed, and cells were gently washed three times with PBS and incubated with PFA 4% for 30′ at room temperature. After incubation, PFA was removed, and cells were washed and rinsed with PBS. For evaluation of the phagosome acidification dependent on α-LA stimulation, uninfected cells were incubated with α-LA at 0.09 mg/mL, 0.075 mg/mL, 0.06 mg/mL, and 0.045 mg/mL. Cells incubated with LPS from E.coli O127:B8 (L4516, Sigma-Aldrich) at a concentration of 2 µg/mL were used as the positive control, while untreated cells were used as the negative control. At 4 h and 24 h post-incubation, live cells were washed and stained as described above. Representative images of each sample were acquired with Cytation5 in brightfield, GFP channel (465 nm LED, Ex 469/35, Em 525/39 Filter Cube). Fluorescence was quantified with Fiji software (ImageJ).

### 4.11. Statistical Analysis

Data collection and analysis were performed using Microsoft Excel (2016) and GraphPad Prism software, version 9.0.0 (GraphPad Software, LLC, San Diego, CA, USA). All experiments were carried out in three independent biological replicates, with each condition tested in triplicate (technical replicates). To account for inter-experimental variability while preserving the representation of natural variance, data were normalized within each independent experiment. Specifically, raw values for both treated and untreated samples were expressed as a ratio relative to the mean of the corresponding untreated control group. This approach allows for visualization of the standard deviation (SD) within the control group itself. The results are presented as the mean ± SD of these normalized values. Statistical significance was assessed by one-way or two-way ANOVA followed by Dunnett’s post hoc tests for multiple comparisons. Statistical significance was defined as follows: ns, *p* > 0.05; *, *p* ≤ 0.05; *p* ≤ 0.01; ***, *p* ≤ 0.001; ****, *p* ≤ 0.0001.

## 5. Conclusions

In conclusion, these combined data suggest that α-LA acts as a multifaceted immunomodulator capable of counteracting Mtb’s main immune evasion strategies by restoring phagosome acidification and promoting apoptotic cell death. While this in vitro model, based on human macrophage cell lines, provides preliminary evidence, further testing and validation in more sophisticated and representative infection models—including cell co-culture systems and animal models—will be necessary to confirm the full therapeutic potential of α-LA in a complex immune system.

## Data Availability

Data are available upon request to the corresponding author.
